# Spinal microglial motility is independent of neuronal activity and plasticity in adult mice

**DOI:** 10.1186/1744-8069-6-19

**Published:** 2010-04-09

**Authors:** Tao Chen, Kohei Koga, Xiang-Yao Li, Min Zhuo

**Affiliations:** 1 Department of Physiology, Faculty of Medicine, University of Toronto Centre for the Study of Pain, University of Toronto, Toronto, Ontario, Canada; 2 Department of Brain and Cognitive Sciences, College of Natural Sciences, Seoul National University, Seoul 151-746, Korea

## Abstract

Microglia are the resident macrophages in the central nervous system. In the spinal cord dorsal horn, microglia stay in resting condition during physiological sensory processing, and are activated under pathological conditions such as peripheral nerve injury. In cases such as this, the nearby resting microglia increase their motility and accumulate at the site of injury. However, direct evidence to support that nerve activity can enhance the motility of microglia has not yet to be reported. In this study we investigated whether the activation of spinal microglia under in vivo nerve injury may be mimicked by neuronal activity in the spinal cord slice preparation. We found that local application of spinal excitatory neurotransmitters, such as glutamate and substance P did not cause any change in the motility of microglial cells in the spinal cord dorsal horn. The motility of microglial cells is unlikely modulated by other transmitters, neuromodulators and chemokines, because similar applications such as GABA, serotonin, noradrenaline, carbachol, fractalkine or interleukin did not produce any obvious effect. Furthermore, low or high frequency stimulation of spinal dorsal root fibers at noxious intensities failed to cause any enhanced extension or retraction of the microglia processes. By contrast, focal application of ATP triggered rapid and robust activation of microglial cells in the spinal dorsal horn. Our results provide the first evidence that the activation of microglia in the spinal cord after nerve injury is unlikely due solely to neuronal activity, non-neuronal factors are likely responsible for the activation of nerve injury-related microglial cells in the spinal dorsal horn.

## Background

Microglial cells are the principal immune-response cells in the central nervous system (CNS) [1,2]. In physiological conditions, they are found in a "resting" state - typically exhibiting ramified processes with high motility [3]. Under pathological conditions, these cells are transformed from the resting condition to an activated condition, exhibiting phagocytoxic, chemotaxis and secretory reactions [4-6]. A growing body of literature indicates that spinal microglia can be activated after nerve injury [4,5], suggesting the possibility that neuronal activity may contribute to microglia activation. This possibility is further supported by studies showing that several neurotransmitter receptors can be expressed on cultured microglia cells, including NMDA, GABA, opioid and adrenergic receptors [7-11]. However, in a recent study using the brain slice preparation for adult mice, we found that microglia did not respond to either a glutamate or GABA application, or activity-dependent long-term potentiation (LTP) [12]. In addition to these findings, we found that nerve injury did not cause any activation of microglial cells in supraspinal central nuclei such as the anterior cingulate cortex (ACC) where excitatory synaptic transmission was significantly enhanced after nerve injury [13]. In support of previous reports in the spinal cord, we also found that microglial cells were activated in spinal cord dorsal horn after the nerve injury [13]. One possible explanation is that spinal microglia may be more sensitive to abnormal neuronal activity than those in higher brain regions.

The spinal cord dorsal horn is a key area for nociceptive transmission and modulation [14]. In the spinal cord, LTP is proposed to be the key cellular mechanism for pathological pain [14,15]. Noxious stimuli of sciatic nerve or hindpaw can induce LTP in spinal dorsal horn neurons [15,16]. Although microglia in the spinal cord is believed to play an important role in neuropathic pain [4,13,17], it is still unclear if LTP inducing protocols activates spinal microglia. To test this possibility, we investigated the motility of microglia in the dorsal horn of spinal cord slices of transgenic mice with green fluorescent protein (GFP) exclusively expressed in microglia [12,18]. Chemicals known to mimic or enhance neuronal activity were applied locally. Electrical stimulation of the dorsal root fibers that has been known to induce spinal LTP was also tested. We found that the activation of the spinal microglia is independent of synaptic or neuronal activity, and the activation after nerve injury is unlikely driven by nerve activity in an activity-dependent manner.

## Results

To investigate the microglial cells in spinal cord dorsal horn, we used transgenic mice with GFP-labeled microglia cells as previously reported [12,18-20]. Similar to microglial cells in supraspinal structures in vivo and in vitro [12,13,20], most of the microglia cells in the spinal cord slices showed ramified with active moving processes, while hypertrophied, monopolarized, bipolarized and amoeboid cells were also observed. These results suggest that the status of microglia in *in vitro *spinal cord slices is similar to those in spinal dorsal horn *in vivo*. Under the confocal microscope, the processes of ramified microglia in brain slices were very dynamic, showing rapid extensions and retractions with a speed of 1 μm/min.

### Effect of excitatory neurotransmitters on the motility of microglia in spinal dorsal horn

**Figure 1:**
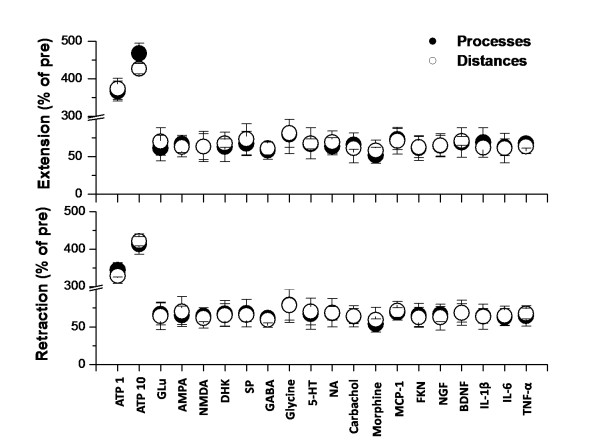
**Summarized effect of different kinds of neurotransmitters, neuromodulators and chemokines on the extension and retraction percentage of microglia in spinal dorsal horn**. The ratio of extended or retracted processes and distances of microglia at time 30 min to 0 min after drug application are shown. ATP are applied at 1 or 10 mM; Glu, AMPA, NMDA, DHK, SP, GABA, Glycine, 5-HT, NA, Carbachol and Morphine are applied at 1 mM; MCP-1, FKN, NGF, BDNF, IL-1β, IL-6 and TNF-α are applied at 10 ng/ml. The results of application of 10 mM Glu, AMPA, NMDA, DHK, SP, GABA, Glycine, 5-HT, NA, Carbachol and Morphine and 100 ng/ml MCP-1, FKN, NGF, BDNF, IL-1β, IL-6 and TNF-α are similar. ATP 1, ATP 1 mM; ATP 10, ATP 10 mM; Glu, Glutamate; DHK, dihydrokainate; NA, noradrenalin; SP, Substance P; MCP-1, monocyte chemoattractant protein-1; FKN, fractalkine; NGF, nerve growth factor; BDNF, brain-derived neurotrophic factor; IL-1β, interleukin-1β; IL-6, interleukin-6; TNF-α, tumor necrosis factor-α.

**Figure 2:**
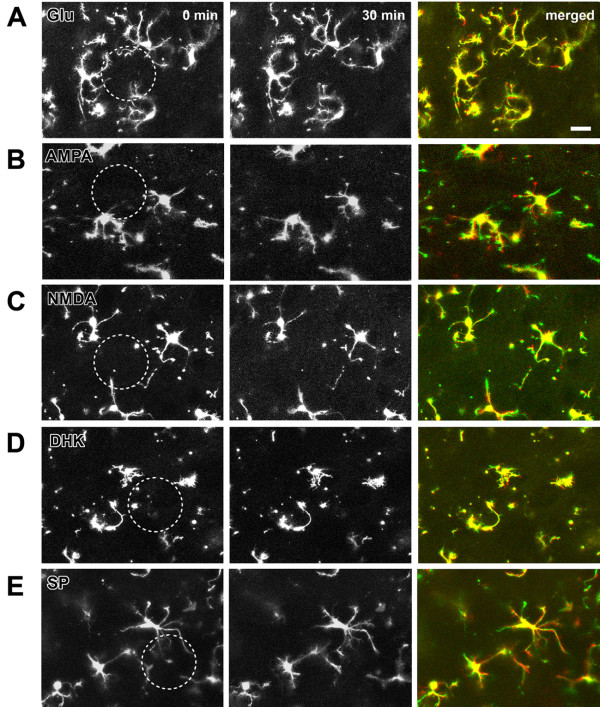
**Effect of excitatory neurotransmitters on the motility of microglia in spinal dorsal horn**. (**A-E**). Local application of 1 mM glutamate (Glu), AMPA, NMDA, glutamate uptake inhibitor dihydrokainate (DHK) or substance P (SP) cannot increase the motility of microglia. Dotted circle indicates the center of the drug application area. Microglial motility was imaged every 2 min for 30 min. The first image (0 min, left) and last image (30 min, middle) were shown here. To observe clearer motility, the 2 images were merged (right) and set as green and red for 0 and 30 min, respectively. Therefore the green reflects retracted portions, and the red reflects extended portions, whereas the yellow reflects unaltered portions. Bars equal to 20 μm.

Glutamate is the major excitatory neurotransmitter in the spinal cord dorsal horn [21]. To test if glutamate may activate spinal microglial cells, we locally applied (1 or 10 mM) glutamate to spinal microglial cells using a picopump application system [12]. We found that glutamate application did not cause any effect on the motility of microglia. No change in the motility of processes, either extension or retraction, was detected (Fig. 1, Fig. 2A). The processes of microglia showed a withdrawal tendency over the time. This is called "rundown" effect in microglial motilities, identified with gradually declined extension and retraction [12]. The ratio of extended/retracted processes or distances of microglia at time 30 min to 0 min after application of glutamate (1 mM, n = 6 slices/5 mice) was shown in Fig. 1. The ratio of extended/retracted process and distances of microglia after 10 mM glutamate application (n = 5 slices/5 mice) is no statistical difference from those in group with 1 mM glutamate application (*p *> 0.05).

We also applied AMPA and NMDA at different doses (1 or 10 mM) to examine if microglial cells may be activated. Similar to the results of glutamate, neither AMPA nor NMDA application affect the status of microglial cells in the spinal cord dorsal horn (n = 4 slices/4 mice in each group; Fig. 1, Fig 2B-C). To examine if the enhancement of local glutamate by inhibiting glutamate uptake may affect microglia, we also applied the selective glutamate uptake inhibitor dihydrokainate (DHK) at 1 or 10 mM. We found that DHK did not cause any obvious effect (n = 4 slices/3 mice; Fig 1, Fig 2D). In addition to glutamate, Substance P (SP) is a neuropeptide that contributes to nociceptive sensory transmission in the spinal cord [22]. To test if SP may trigger any activation of microglia, we performed local application of SP at two different doses (1 or 10 mM). Similarly, SP did not induce any obvious enhancement in the motility of microglia (n = 5 slices/5 mice in each group; Fig. 1, Fig 2E).

### Effect of inhibitory neurotransmitters on the motility of microglia in spinal dorsal horn

**Figure 3:**
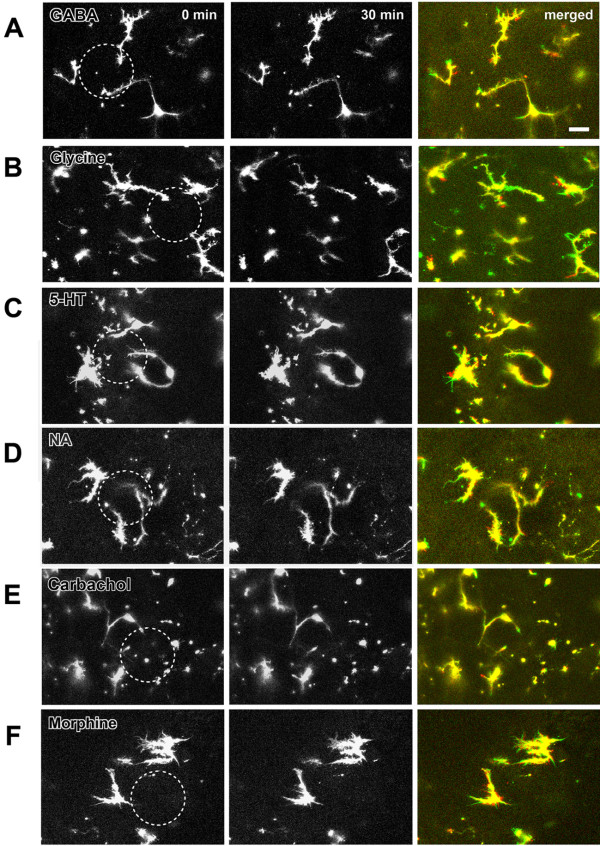
**Effect of inhibitory neurotransmitters and neuromodulators on the motility of microglia in spinal dorsal horn**. (**A-F**). Local application of 1 mM GABA, Glycine, 5-HT, noradrenalin (NA), acetylcholine agonist carbachol and morphine had no effect on microglia motility. The merged picture is the overlay of imaging at 0 min (green) and 30 min (red) after drug local application. Note that (**F**) showed the result recorded on typical amoeboid cells. Bars equal to 20 μm.

GABA and glycine are two major inhibitory transmitters in the spinal cord dorsal horn. If microglia can be regulated by synaptic activity, we expect that application of GABA or glycine may reduce the motility of microglia. After local application of all of these drugs (1 or 10 mM), no significant change in motility was observed (Figs. 3A-B). The ratio of extended/retracted processes and distances of microglia at time 30 min to 0 min after application of 1 mM GABA (n = 6 slices/6 mice) or glycine (n = 5 slices/4 mice) were summarized in Fig. 1. The ratio of extended/retracted process or distances of microglia after 10 mM GABA (n = 5 slices/5 mice) or glycine (n = 5 slices/4 mice) application is not statistically different from those with 1 mM GABA or glycine application (*p *> 0.05). These results suggest that enhancing spinal inhibitory transmission did not affect the motility of microglia.

### Effects of neuromodulators on the motility of microglia in spinal dorsal horn

Spinal nociceptive transmission is regulated by multiple neurotransmitters released locally or from descending projection fibers, including serotonin (5-HT), noradrenalin (NA), acetylcholine and opioid peptide [23-27]. Because neither excitatory transmitter glutamate nor inhibitory neurotransmitter GABA/glycine enhance the motility of microglia, we decided to examine whether neuromodulators have affect on the motility of microglia. Agonists that activate the neuromodulators' receptors, including 5-HT, NA, carbachol and morphine, were applied at two different doses (1 or 10 mM). After application of 1 mM 5-HT (n = 4 slices/4 mice), NA (n = 5 slices/4 mice), carbachol (n = 5 slices/4 mice) or morphine (n = 6 slices/5 mice), however, no significant enhancement of the motility of microglia was observed (Figs. 3C-F). The ratios of extended/retracted processes or distances of microglia were summarized in Fig. 1. The ratios of extended/retracted processes or distances after 10 mM drug application were similar to those after drug application (*p *> 0.05).

### The effect of the stimulation of dorsal root on the motility of microglia

**Figure 4:**
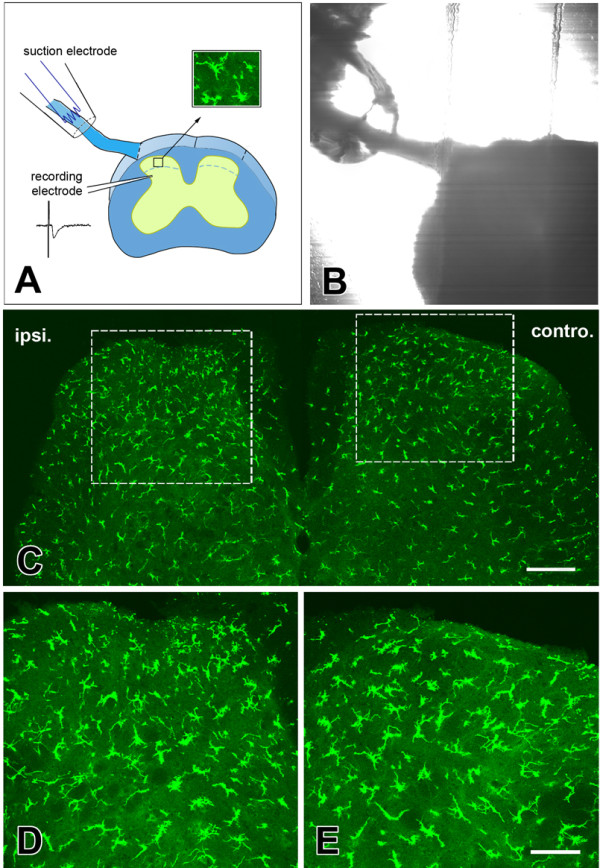
**Histological results of dorsal root stimuli on the motility of microglia in spinal dorsal horn**. (**A**). Schematic representation of the confocal imaging and field potential recording accompany with dorsal root stimuli; (**B**). Digitized photomicrographs of the dorsal root stimuli model in a transverse spinal cord section. (**C**). Observation of microglia in one fixed section of spinal cord after low frequency dorsal root stimuli. Rectangled areas in (**C**) were magnified in (**D**) and (**E**) respectively. Ipsi., ipsilateral part of dorsal root; Contro., controlateral part of dorsal root. Bars equal to 100 μm in (**C**) and 50 μm in (**D**) and (**E**).

**Figure 5:**
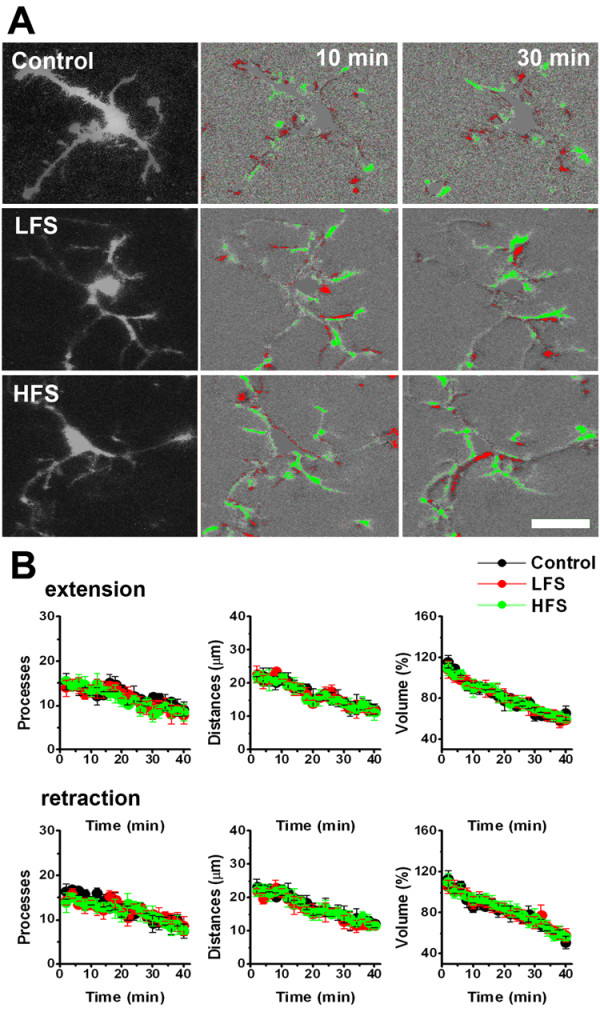
**Effect of low or high frequency stimuli to the dorsal root on the motility of microglia in spinal dorsal horn**. (**A**). Sample images showing the extension and retraction of microglia in control (top), low frequency stimulation (LFS) (middle) and high frequency stimulation (HFS) induction groups (bottom). Right image is showing the microglia at 10 min and 30 min after imaging. LTP induction was delivered at time 10 min. (**B**). Extension (top) and retraction (bottom) of microglia in LTP induction groups is similar to that in control group. The number, distance, and percentage of volume of extension or retraction processes were measured. Note that the "rundown" of microglial extension was found in the three groups.

Activity-dependent synaptic plasticity is thought to be important for the formation of hyperalgesia, in which microglia is believed to be activated and play important roles [4,28-30]. In the spinal cord dorsal horn, it's reported that two kinds of stimulation patterns (low frequency stimulation (LFS) and high frequency stimuli (HFS)) can induce LTP effect in different portion of neurons [15,28,29]. Thus, we decided to test the effects of LFS and HFS on the motility of spinal microglia. LFS or HFS was delivered by a suction electrode at an intensity sufficient to activate Aδ and C fibers (Fig. 4). To study the extension and retraction of microglia processes, the number and distance of extending and retracting microglial processes was calculated. Furthermore, the percentage of the extension or retraction volume 10 min before and 30 min after the LFS and HFS induction were calculated. Fig. 5A shows one sample from control, LFS and HFS group respectively at time 10 min and 30 min. Data is summarized in Fig. 5B. Six microglia from six separate slices were counted in each group, and a similar rundown of microglial motilities were found during and after stimuli were applied. No significant difference in microglial motilities, such as the number (9.0 ± 1.6, 7.8 ± 2.0 or 8.4 ± 2.0 in control, LFS or HFS group, P >0.05), distance (μm) (12.1 ± 1.6, 11.8 ± 0.9 or 11.1 ± 2.2 in control, LFS or HFS group, P > 0.05), or volume (%) (66.0 ± 6.7, 59.4 ± 2.3 or 60.5 ± 4.2 in control, LFS or HFS group, P > 0.05) of extension were observed. Similarly, there was no significant difference in all of these parameters of retraction before and after LFS or HFS (*P *> 0.05) (Fig. 5B).

Peripheral nerve injury can also increase the number of microglia in spinal dorsal horn [5,13,17]. If short time stimulation (like LTP induction) on the dorsal root can activate microglia, it may also cause the similar results on spinal cord slices. The more likely effect should be microglia cells redistributed, in which microglia in the middle part of the spinal cord may be attracted by, and approach close to, the dorsal root. To test this proposal, we fixed the spinal cord slices after LFS or HFS, then checked the number and distribution pattern of microglia in spinal cord slices. The number of the microglia bodies in 30 μm sections in layers I-V ipsilateral or contralateral to the dorsal root stimulation site was calculated and compared. After LFS or HFS, we found that the number of microglia in different layers, as well as the morphology of microglia were not significantly affected (P > 0.05, n = 6 mice in each group) (Table 1 and Table 2).

**Table 1:** Number of microglia cells in layer I-V of spinal dorsal horn after low frequency dorsal root stimuli (n = 6)

Lamina	Ipsilateral (number/mm^2^, Mean ± SEM)	Contralateral (number/mm^2^, Mean ± SEM)
I	314.5 ± 19.4	301.5 ± 12.8
II	289.7 ± 15.3	273.1 ± 16.4
III	212.1 ± 22.3	226.2 ± 17.8
IV	180.5 ± 10.8	165.4 ± 17.2
V	182.3 ± 16.7	183.8 ± 14.3

**Table 2:** Number of microglia cells in layer I-V of spinal dorsal horn after high frequency dorsal root stimuli (n = 6)

Lamina	Ipsilateral (number/mm^2^, Mean ± SEM)	Contralateral (number/mm^2^, Mean ± SEM)
I	331.5 ± 22.3	311.1 ± 16.7
II	290.7 ± 18.4	293.9 ± 17.7
III	224.3 ± 23.9	242.0 ± 18.8
IV	197.1 ± 18.2	177.0 ± 16.3
V	190.8 ± 14.5	182.7 ± 10.9

### Effect of chemokines on the motility of microglia in spinal dorsal horn

**Figure 6:**
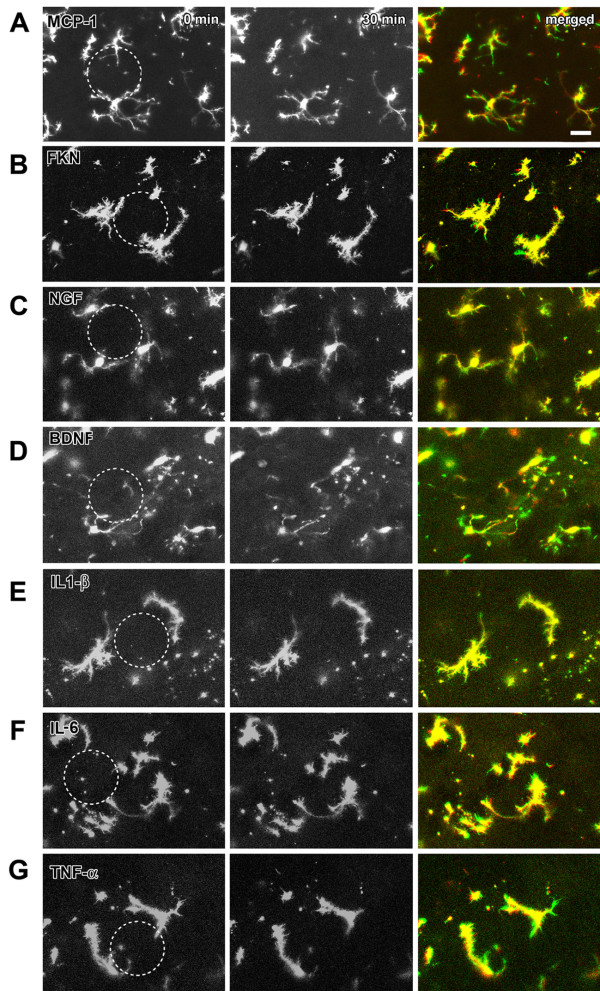
**Effect of chemokines on the motility of microglia in spinal dorsal horn**. (**A-G**). Local application of 10 ng/ml monocyte chemoattractant protein-1 (MCP-1), fractalkine (FKN), nerve growth factor (NGF), brain-derived neurotrophic factor (BDNF), interleukin 1β (IL-1β), interleukin 6 (IL-6) or tumor necrosis factor α (TNF-α) had no effect on microglia motility. The merged picture is the overlay of imaging at 0 min (green) and 30 min (red) after drug application. Figs. (**E-G**) showed the results recorded on amoeboid cells. Bars equal to 20 μm.

Chemokines, especially MCP-1 and FNK, have been suggested to contribute to microglia activation [4,31,32]. However, in the present study, we found that application of MCP-1 or FNK did not affect the motility of microglia in the spinal cord slices (Figs. 6A-B). The ratios of extended/retracted processes or distances of microglia at time 30 min to 0 min after application of 10 ng/ml MCP-1 (n = 6 slices/5 mice) or FNK (n = 5 slices/5 mice) were summarized in Fig. 1. Similar results were also found with 100 ng/ml MCP-1 or FNK.

In spinal cord dorsal horn, activated microglia can release some chemokines like NGF, BDNF, IL-1β, IL-6 and TNF-α. These factors may spread and activate adjacent microglia and neurons [4,5,17,33]. To test the possible effects on spinal microglia, we applied them (10 or 100 ng/ml) to the microglia cells in spinal cord slice preparation. Application of NGF (n = 4 slices/3 mice), BDNF (n = 5 slices/4 mice), IL-1β (n = 6 slices/6 mice), IL-6 (n = 5 slices/4 mice) or TNF-α (n = 6 slices/5 mice) did not enhance the motility of the microglia (Figs. 6C-G). The ratios of extended/retracted processes or distances of microglia after application of 10 ng/ml NGF, BDNF, IL-1β, IL-6 or TNF-α were summarized in Fig. 1.

### Effect of ATP on the motility of microglia in spinal dorsal horn

**Figure 7:**
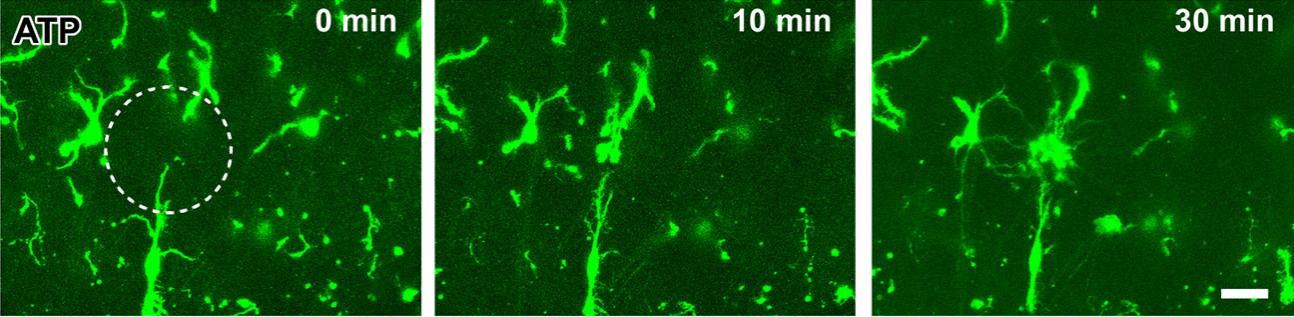
**Effect of ATP on the motility of microglia in spinal dorsal horn**. Local application of ATP (1 mM) induced rapid movement of microglial processes toward the tip of puff pipette in acute spinal cord slices. Three time points of 0 min, 10 and 30 min after ATP application were shown here. Bar equals to 20 μm.

ATP is known to be a key regulator for microglia during the injury. Microglia expresses both ionotropic (P2X) and metabotropic (P2Y) ATP receptors. Under both in vivo and in vitro brain slice conditions, it has been reported that ATP induced an obvious enhancement of the motility of microglia and caused a chemotaxis effect [3,12,19,20]. In the present study, we wanted to examine if similar results can be found in spinal cord slices. Application of ATP at a low (1 mM) or a high (10 mM) concentration induced obvious effects on the motility of microglia. Microglia stretched out with terminals moving towards the site of the injection pipette. The microglia terminals extended toward the tip of the pipette with an average velocity of 0.8 ± 0.3 μm/min (1 mM, n = 12) or 1.4 ± 0.2 (10 mM, n = 14). At 20-30 min after the injection, the processes reached the site, forming a spherical shield around it (Fig. 7). The ratio of extended processes and distances at time 30 min to 0 min after application of 1 mM (n = 5 slices/4 mice) or 10 mM (n = 6 slices/4 mice) ATP were shown in Fig. 1.

## Discussion

In this study, by combining a time lapse confocal imaging and histological observation, we demonstrated that motility of microglia in the spinal dorsal horn is not driven by neuronal activity directly. Stimulation of sensory afferent fibers at noxious intensities did not cause any activation of microglial cells. Furthermore, stimulation protocols that are known to induce long-term plasticity in spinal cord dorsal horn neurons failed to trigger any alteration in microglia. These results are important as they provide the first evidence that spinal microglia are not likely regulated by sensory inputs under physiological or pathological conditions. Although recent studies confirmed the activation of spinal microglia by nerve injury using the same transgenic mice [13], we suggest that activation of microglia in animal models for neuropathic pain is likely caused by non-neuronal factors after nerve injury. However, we cannot rule out the possibility that the microchemical environment within spinal microglial cells may change and release chemical, diffusible molecules.

In physiological conditions, the majority of microglia are found to be in a resting state. They have ramified processes with continuous extending and retracting reaction for detecting changes [3,19]. Our previous work of cortical and hippocampal slices confirm the existence of similar resting microglial cells within the central nervous system. In animal models of neuropathic pain in rats and mice, activation of microglia were consistently observed as discussed in our recent paper. One major hypothesis is that the activation of microglia by nerve injury is caused by abnormal sensory input activity. However, in the present study, we found that microglial cells were not activated by nerve activity or application of different chemicals that mimic sensory transmission and modulation. One possible explanation for this failure to activate is the use of in vitro slice preparation. Our recent studies however favor the use of brain/spinal slice preparation for the investigation of microglia [12,20,34]. In all slice preparations including the present study, we found that the basal microglia are similar to in vivo resting microglia in the same brain region, and application of ATP but not Glu and GABA can enhance the motility of microglia [12,20].

Despite the expression of AMPA, NMDA, GABA, opioid and adrenergic receptors on cultured microglia [7-11], the present study showed no effect of different receptor agonists on the resting microglia in acute spinal cord slices. We propose that microglia in CNS may not be 'simply' activated by neuronal activity or LTP/LTD like plasticity.

Chemokines are one kind of signal involved with microglia activation. Among them, MCP-1 and FKN are considered as neuronal-microglia signals. They are reported to be expressed on DRG neurons and spinal dorsal horn neurons after nerve injury [35-39]. MCP-1 and FKN can combine to their receptors [4,31,32] expressed on microglia and induce their activation. Consequently, activated microglia release chemokines like BDNF, NGF, IL-1β, IL-6 and TNF-α, which act on resident microglia and neurons and result in continuous tissue sensitization and exaggerated pain [6,40-42]. In this study, we didn't find any enhanced motility of microglia after local application of MCP-1, FKN, BDNF, NGF, IL-1β, IL-6 and TNF-α, indicating that a single chemokine is not powerful enough to enhance the motility of microglia in spinal cord slices.

In the present study, ATP application but not dorsal root stimulation can cause increased motility of microglia. This finding confirms that microglial cells in spinal cord slice preparation are similar to those in vivo in term of ATP sensitivity. Considering ATP can be released from efferent terminals after dorsal root stimulation [9], we think that removing the possible inhibitory elements in the circumstance context in pathological condition may be important for the activation of microglia. In normal condition, dorsal root stimulation induced synaptic release of ATP may be rapidly degradation by ATP-hydrolyzing enzyme in situ and cannot reach a high degree to increase the motility of microglia [9].

Although no enhanced motility of microglia was observed, it does not exclude the possibility that biochemical changes may happen after drug application or LTP induction within microglial cells. After peripheral nerve injury, the first changes in spinal microglia are the withdrawal processes and enlarged soma [5], which is similar to the "rundown" phenomenon observed in our study and reflects a gradual activation of microglia. Since the increased number of microglia in spinal cord only reach the peak 2-3 days after nerve injury, the neuronal activity and LTP induction induced activation of microglia may be a slowly procedure [5,13]. In current study, thirty minutes observation may be not long enough for detecting possible chronic activation of microglia, long-term observations under in vivo conditions should be carried out in the future to study if microglia in spinal cord can be activated after dorsal root stimulation.

In summary, our work on the spinal cord dorsal horn indicates that the motility of spinal microglia cannot be enhanced by dorsal root stimuli or local application of neurotransmitters, neuromodulators and chemokines, which is consistent with our previous works on brain slices. These results suggest that resting microglia in the CNS may not be directly related to synaptic transmission and plasticity. Activation of microglia in a cultured dish or *in vivo *may be due to the combined effects of the co-release of several signals and removal of inhibitory factors.

## Methods

### Transgenic mice

Heterozygous Cx3cr1^GFP/+ ^mice were used for all the experiments [18]. All mice were maintained on a 12-h light/dark cycle with food and water provided ad libitum. All protocols used were approved by The Animal Care and Use Committee at the University of Toronto.

### Acute spinal cord slice preparation

Adult male or female mice (6-10 wks old) were anesthetized with 1-2% urethan, and body temperature was kept in the range 35-37°C with an infrared lamp. A lumbosacral laminectomy was performed and a 1-2.0 cm length of spinal cord with attached dorsal roots was excised. The spinal cord was placed in cold (0-4°C) oxygenated (95% O_2_-5% CO_2_) artificial cerebrospinal fluid (ACSF) solution containing (in mM) 124 NaCl, 25 NaHCO3, 2.5 KCl, 1 KH_2_PO_4_, 2 CaCl_2_, 2 MgSO_4_, and 10 glucose. After removal of the dura mater, all ventral and dorsal roots, with the exception of the L3 or L4 dorsal root on one side, were cut near the root entry zone. The pia-arachnoid membrane was then removed, sparing the area around the site of insertion of the preserved dorsal root. The spinal cord, together with the attached dorsal root, was placed in a shallow groove formed in an agar block. The spinal cord was immersed in cold ACSF solution and a transverse slice (500-700 μm) with attached dorsal root was cut with a Vibratome according to the protocols reported before [43,44]. The slice was then completely submerged and superfused continuously with ACSF solution equilibrated with 95% O_2_, 5% CO_2 _at room temperature for 1 hour and then transferred to a recording chamber and perfused with oxygenated ACSF solution at 3-4 ml/min at 34 ± 1°C. The dorsal root of the spinal cord slice was then sucked into a suction electrode for electronic stimuli.

Spinal cord slices without dorsal root were also prepared for local drug microinjection. All the procedures for making slices are the same to those mentioned above except that all the dorsal roots and pia-arachnoid were thoroughly removed from the surface of the spinal cord and spinal cord slices without dorsal roots were cut. Local drug application and LTP induction were carried out in slices within 3 hrs after cutting operation for observing the motility of ramified microglia and in slices more than 3 hrs to observe the motility of ameboid microglia cells.

### Confocal imaging

GFP-labeled microglia in dorsal horn of the spinal cord (layers I-V) were imaged by confocal microscope (Fluoview FV 1000, Olympus, Tokyo, Japan). The laser with a wavelength of 488 nm was used for GFP excitation, and 633 nm was used for DIC images. The image of microglia was collected for 8-10 consecutive focal steps of 2 μm once every 2 min using × 40, 0.8 numeric aperture water-immersion objectives. XYZT image galleries were collected, and Z projections were made for the quantification.

### Local Drug microinjection procedures

All drugs were obtained from Sigma (St. Louis, MO, USA) except monocyte chemoattractant protein-1 (MCP-1), fractalkine (FKN), nerve growth factor (NGF), brain-derived neurotrophic factor (BDNF), interleukin (IL)-1β, IL-6 and tumor necrosis factor (TNF)-α (Peprotech, Embrun, Canada). A picopump (pneumatic picopump, WPI, Sarasota, FL) was used to apply neurotransmitters, neuromodulators and cytokines to induce microglial chemotaxis according to our previous reports [12]. The diameter of the drug application pipette tip was 3-4 μm. The pressure and duration of the puff was 5 psi and 100 ms, respectively.

### Recording and stimulation

To study the effect of electronic stimuli on microglial motilities, the 40 volt, 0.5 ms low frequency (LFS, 2 Hz, 2-3 min) or high frequency (HFS, 100 Hz, given in 5 trains of 1 s duration at 10 s intervals) electronic stimuli, which were proved to induce LTP effect in different groups of spinal dorsal horn neurons, were delivered through the electrode [15,28,29]. To make sure that the stimuli could be carried out properly, a field recording electrode was used for field excitatory postsynaptic potential (EPSP) recording. The recording electrode was filled with ACSF and inserted into ipsilateral part of the layer V-VI, 200-300 μm far away from the confocal imaging area. The field EPSP data was amplified and filtered by Axopatch 200B amplifier, digitalized by Digidata 1322A and analyzed by pClamp 8.0 software (Molecular Devices, Union City, CA).

### Histology

After the procedure for dorsal root stimuli and confocal imaging, spinal cord slices with dorsal root would be kept staying in ACSF for one hour and then removed to fixation solution containing 4% paraformaldehyde in 0.01 M phosphate buffered saline (PBS, pH 7.4) for 24 hrs. Subsequently, the spinal cords were placed into 30% (w/v) sucrose in 0.01 M PBS overnight at 4-8°C. After making a pinhole in the ventral part of the sample contralateral to the dorsal root, the fixed spinal cord was cut serially into 30 μm-thick frontal sections on a freezing microtome (Kryostat 1720; Leitz, Mannheim, Germany). Then the serial sections were mounted onto glass slides, air dried, cover-slipped with a mixture of 50% (v/v) glycerin and 2.5% (w/v) triethylene diamine in 0.01 M PBS, and observed with the confocal microscope (FV 1000; Olympus).

### Data analysis

The microglial motilities were analyzed by using Image-Pro Plus software (IPP 5.0., Media cybernetics, Silver Spring, USA). The number, distance, and volume of extending and retracting microglial processes were calculated every 2 min. Microglia quantification was performed on sections of 30 μm thickness. Only the cell bodies were taken into account. Cell counting was performed by using confocal microscope under 20× objective through IPP 5.0 software. Layers I-V in ipsilateral and controlateral sites of dorsal root were examined for each of the three randomly selected sections per mouse. Data were expressed as mean ± SEM. Statistical comparisons were performed with the Student's t-test. In all cases, P < 0.05 was considered statistically significant.

## Competing interests

The authors declare that they have no competing interests.

## Authors' contributions

TC carried out electrophysiological, imaging and histological experiments and drafted the manuscript. KK participated in electrophysiological and histological experiments. XYL helped with imaging experiments. MZ designed and finished the final draft of the manuscript. All authors read and approved the final manuscript.
